# Supplementary material for: Risk mapping for COVID-19 outbreaks in Australia using mobility data
Source: J R Soc Interface. 2021 Jan 6;18(174):20200657. doi: 10.1098/rsif.2020.0657 (PMC7879754; doi:10.1098/rsif.2020.0657)
Supplement: Supplementary Information [file rsif20200657supp1.pdf]

# Supplementary Information for:

# Risk mapping for COVID-19 outbreaks in Australia using mobility data

Cameron Zachreson,[1] Lewis Mitchell,[2] Michael J. Lydeamore,[3,4]
Nicolas Rebuli,[5] Martin Tomko,[6] and Nicholas Geard[1,7]

[1]*School of Computing and Information Systems, The University of Melbourne, Australia*

[2]*School of Mathematical Sciences, The University of Adelaide, Australia*

[3]*Victorian Department of Health and Human Services, Government of Victoria, Australia*

[4]*Department of Infectious Diseases, The Alfred and Central Clinical School, Monash University, Melbourne, Australia*

[5]*School of Public Health and Community Medicine, University of New South Wales, Australia*

[6]*Melbourne School of Engineering, The University of Melbourne, Australia*

[7]*The Peter Doherty Institute for Infection and Immunity, The Royal Melbourne Hospital and The University of Melbourne, Australia*

## S1. DESCRIPTION OF MOBILITY DATA

The data used in our study was provided by the Facebook Data for Good program. The data set (in the Disease Prevention Maps subset) is aggregated from individual-level GPS coordinates collected from the use of Facebook's mobile app. Therefore, the raw data is biased to over-represent the movements of any subpopulations more likely to utilise social media applications on mobile devices. After collection, the data is spatially and temporally aggregated as a list of trip numbers between Bing Tiles [1] within a rectangular raster pattern (i.e., centered on a country, state, or city). The sizes and boundaries of these discrete locations are determined by an optimisation procedure that produces the smallest subregion size possible (down to a minimum size of 600m $\times$ 600m), given the extent of the region of interest and the requirement for near-real time release of new data. A trip between locations is defined based on the most frequently visited tile in the first 8-hour period and the most frequently-visited tile in the subsequent 8-hour period. Finally, before the data is released, any entries showing fewer than 10 trips between a pair of locations are removed to protect the privacy of individual users. For Australia, the state-level data consists of trip numbers between 2km $\times$ 2km tiles. By comparing this scale to larger (national-scale) and smaller (city-scale) regions of interest, we determined that the state-level data provided the best balance, with trip numbers large enough to produce a sufficiently dense network of connections while still providing a subregion size that is usually smaller than the Local Government Areas for which case data is reported.

### A. Generating correspondences

Because the raw mobility data is provided as movements between tiles, while case data is provided based on the boundaries of Local Government Areas. We note that while Facebook releases data aggregated to administrative regions, these regions were not geographically consistent with the current LGA boundaries for Australia. In order to ensure consistency of our method across datasets and jurisdictions, we produced our own correspondence system. We did this by performing two spatial join operations. These associate either tiles or LGAs with Meshblocks (the smallest geographic partition on which the Australian Bureau of Statistics releases population data). Meshblocks were associated based on their centroid locations. Each meshblock centroid was associated to the tile with the nearest centroid and to the LGA containing it. We did not

split meshblocks whose boundaries lay on either side of an LGA or tile boundary, as their sizes are sufficiently small that edge effects are negligible (in addition, the set of LGAs forms a complete partition of meshblocks, so edge effects were only observed for tile associations). We then associated tiles to LGAs proportionately based on the fraction of the total meshblock population within that tile that was associated with each overlapping LGA.

### B. Re-partitioning mobility data

Once a correspondence is established between the tile partitions on which mobility data is released and the LGA partitions on which case data is released, the matrix of connections between tiles must be converted into a matrix of connections between LGAs. The Supplementary Technical Note explains how we performed this step, and gives a general method for converting matrices between partition schemes. Briefly, the number of trips between two locations in the initial data is split between the overlapping set of partitions in the new set of boundaries (in this case, local government areas), based on the correspondence between partition schemes determined as explained in the previous subsection.

### C. Spatial biases in Facebook mobility data

To investigate the spatial sample biases present in the mobility data provided by Facebook, we examined the ratio of Facebook users to ABS 2018 population for each suburb in Victoria. While the true number varies from day to day, an example of this distribution is shown as a heat map in Supplemental Figure S1, which displays the average number of Facebook mobile app users indexed to each LGA between the hours of 2am and 10am from May 15th to June 25th, divided by the estimated resident population reported by the ABS in 2018. The distribution is narrow, with most urban areas falling in the range of 5 % to 10 % Facebook users. However, this is not an exact representation of residential population proportions, as many mobile users work during the nighttime and will not be located at their residence during the selected period. Unfortunately, it is not possible to precisely quantify the bias introduced by Facebook's sampling scheme.

Despite these limitations, it may still be informative to examine whether accounting for the bias pictured in Figure S1 affects our validation. To determine this, we re-computed the correlations

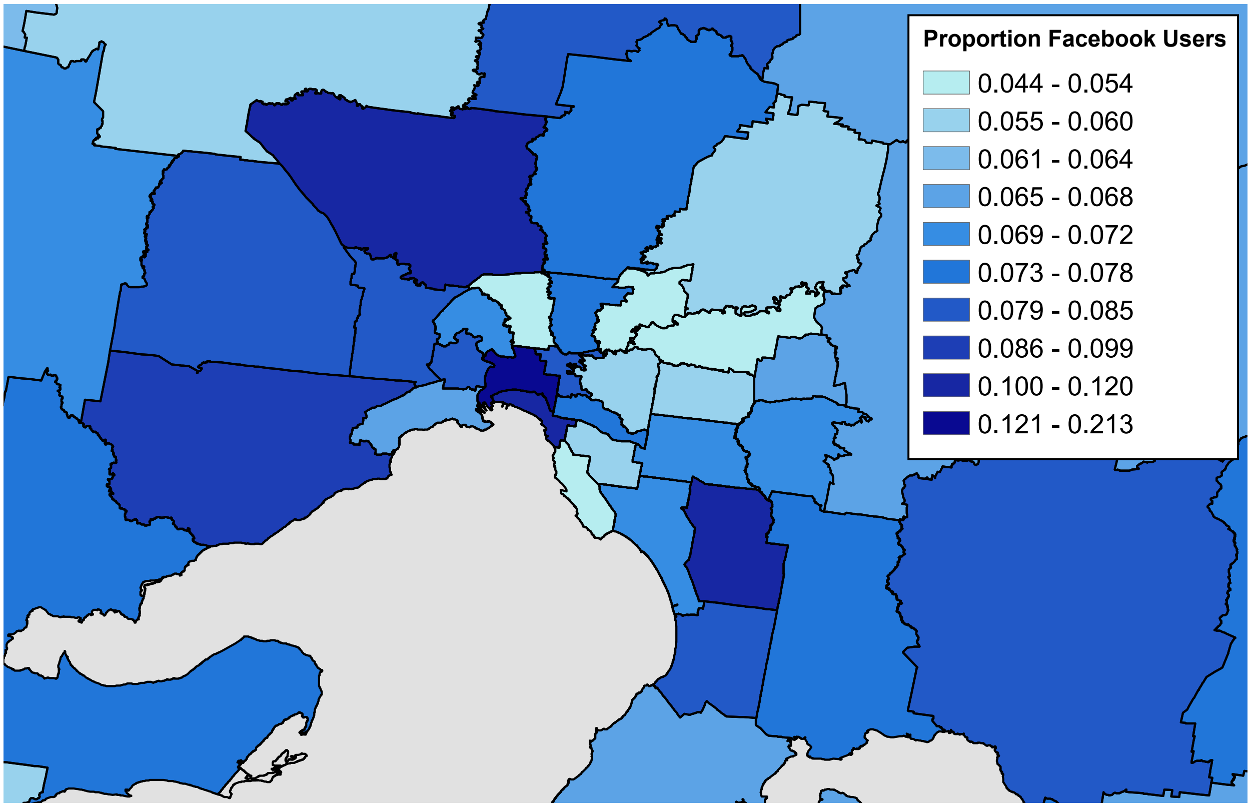


FIG. S1. Heat map of the proportion of Facebook users estimated for each LGA. The values are computed as the number of Facebook users who were found in each location during the hours of 2am until 10am averaged from May 15th to June 25th, divided by the residential population recorded by the ABS in 2018. The colour scale was generated using the method of Jenks natural breaks.

pictured in Figures 3(a) and 5(b) (corresponding to the Cedar Meats and Victoria community transmission scenarios). To do so, we multiplied all mobility flows out of each region by the inverse proportion of Facebook users to the total number of residents in the origin location. For the reasons discussed above, this is not an exact accounting for sample bias, but may partially correct for heterogeneity in the proportion of travellers counted in mobility data released by Facebook.

For the Cedar Meats outbreak scenario, accounting for the Facebook sample bias in this way improves the correlation between our mobility-based relative risk estimate and the recorded case counts (Figure S2a). For the community transmission scenario, performing this extra step does not appear to substantially change the result shown in Figure 5 (compare Figure S2b and Figure 5c).

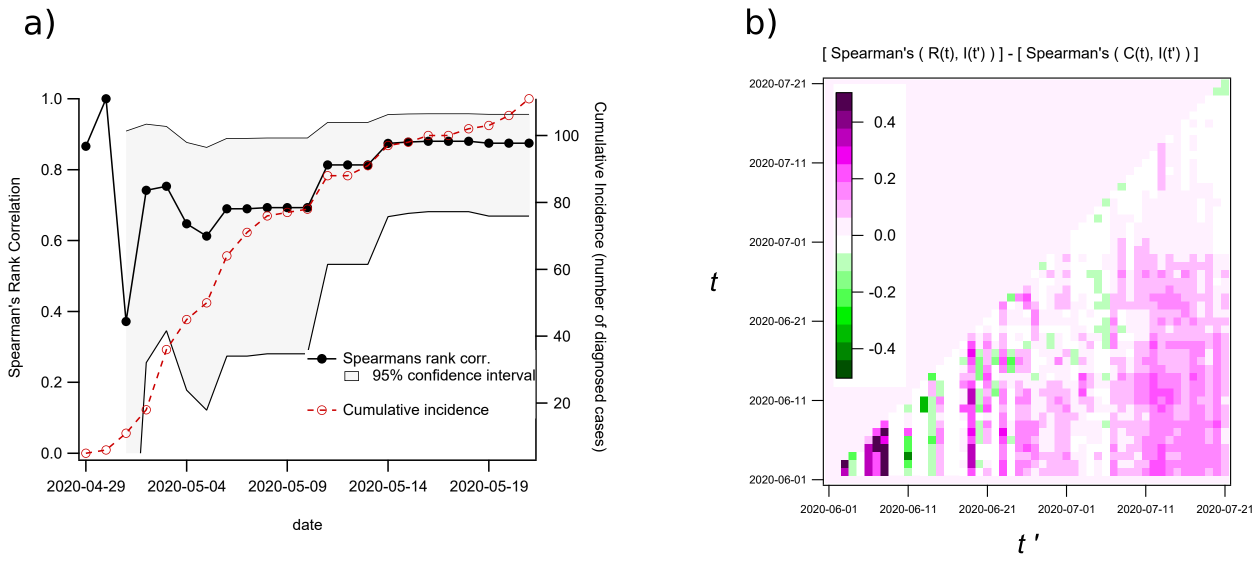


FIG. S2. Correlations between partially bias-corrected risk estimates and documented case numbers. Spearman's rank correlation between cumulative case numbers by LGA and the modified relative risk estimate for the Cedar Meats outbreak is shown in (a). Subfigure (b) shows the partially bias-corrected version of Figure 5(c), demonstrating the effect of including bias-corrected mobility with active case numbers at time $t$ in estimation of relative incident case risk at time $t'$.

### D. Temporal autocorrelation of mobility matrices

In order to investigate the degree to which mobility changed during the study period, we computed the autocorrelation of mobility flows between origin-destination pairs at time $t$ to those at future times $t'$. The results, shown in Figure S3, demonstrate that while weekend and weekday mobility differ markedly, and the implementation of stage 3 restrictions in Greater Melbourne altered mobility patterns, there is a very high level of temporal consistency in relative mobility volumes throughout the studied period. For this reason, our results for the community transmission scenario shown in Figure 5 are robust to the precise selection of time periods used to generate the mobility matrices for our risk estimates. For example, if we integrate mobility flows over a period longer than one week, or consider a nonzero delay between the period over which mobility is averaged and the time $t$ for which active case data is tabulated, it has no effect on the resulting risk rankings and gives the same pattern of temporal correlations (though the risk values themselves are affected slightly).

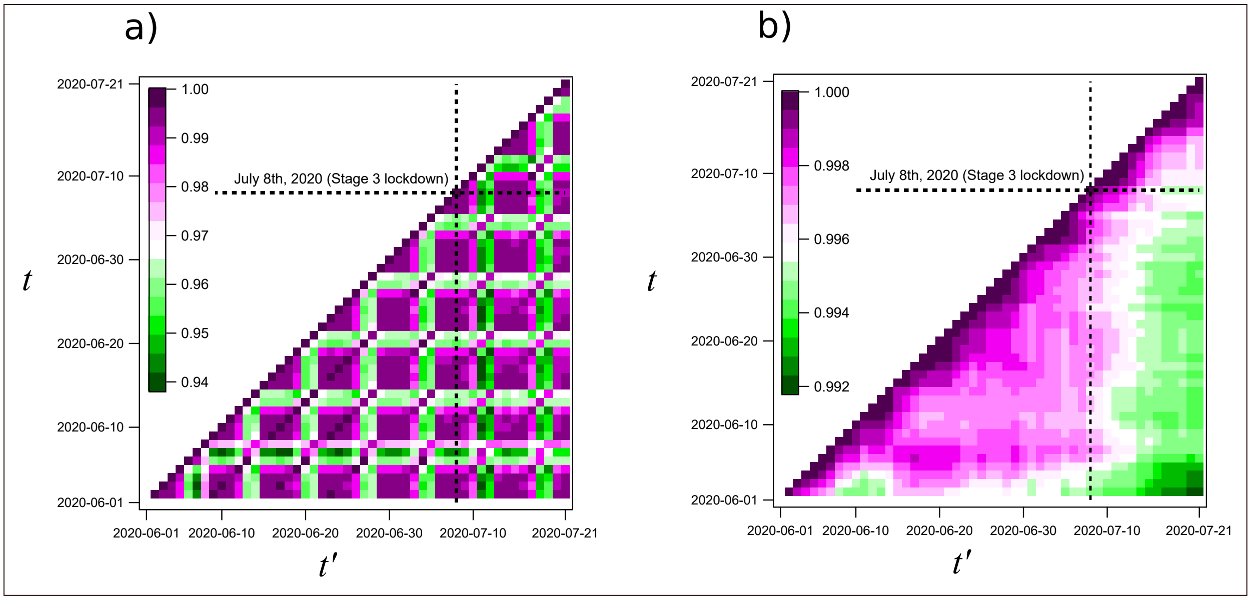


FIG. S3. Autocorrelation (Spearman's rho) of mobility flows at time $t$ (y-axis) and $t'$ (x-axis), for all LGA origin-destination pairs in Victoria, between June 1st and July 21st, 2020. Subfigure (a) shows the raw autocorrelation values, and (b) shows the autocorrelation of the 7-day average. The black dashed lines indicate the date on which Stage 3 distancing restrictions were implemented in the regions of Greater Melbourne and Mitchell Shire.

## S2. CORRELATING RISK ESTIMATES TO CASE DATA

We used Spearman's rank correlation to investigate the correspondence between our relative risk estimates and documented case data. This measure of correlation is typically used when comparing ordinal data, or, more generally, when monotonic relationships are expected, but errors are not normally-distributed. In order to investigate the monotonicity between relative risk estimates and reported case numbers, we aligned the documented case data for all regions in which infections had been tabulated against the corresponding relative risk estimates for those regions. Note that our correlations did not include regions for which no case data was available. Therefore, our correlation results illustrate the degree to which risk estimates are monotonic with case numbers, but do not account for any risk estimates made in areas with no cases to compare to. This results in a high degree of uncertainty when the number of affected areas is small, reflected by the wide confidence intervals observed in the early stages of the Cedar Meats and Crossroads Hotel outbreaks (Figures 3, and 4a, respectively).

The 95% confidence intervals were computed using Fisher's Z transformation with quantile parameter $\alpha = 1.96$.

## S3. ABS DATA SOURCES

Two data sets from the Australian Bureau of Statistics were used in this study: 1) number of residents by industry of occupation (2016), and 2) resident population (2018).

### A. Population by LGA

The distributions shown in Figure S1 were computed by dividing the number of Facebook users indexed to each LGA during the nighttime period by the resident population in each LGA. We obtained the population data from the ABS 2018 population dataset which is publicly available [2]. The Facebook user populations are provided by the Data For Good program in addition to the mobility data discussed above.

### B. Employed persons by industry of occupation

As a context-specific risk factor for the Cedar Meats outbreak we obtained the number of individuals by place of usual residence and industry of occupation. Specifically, we obtained the number of residents in each Local Government Area (2016 boundaries), employed in the occupation categories "Meat Boners and Slicers and Slaughterers" and "Meat Poultry and Seafood Process Workers". This data from the 2016 Australian Census of Population and Housing is available from the ABS TableBuilder web application [3]. We used a population-weighted correspondence list to convert the data provided on geospatial boundaries of 2016 Local Government Areas into 2018 Local Government Area boundaries. For the Melbourne region in which this data was applied, these boundaries have not changed substantially between 2016 and 2018.

To compute the factors used to weight the mobility-based relative risk predictions, we divided the total number of workers in both of the above categories by the number of employed persons (those employed full time or part-time) in each LGA, which we also drew from the 2016 Australian Census via Census TableBuilder.

## S4. CASE DATA

COVID-19 case data by local government area is available from Australian jurisdictional health authorities. For this work, we used data provided by NSW Health [4] (all data is publicly available)

and from Victoria DHHS. The data used for the Cedar Meats outbreak scenario was obtained from DHHS through a formal request to the Victorian Agency for Health Information (VAHI) and cannot be made public in this work. The case data by LGA used to evaluate the Victoria community transmission scenario was taken directly from the COVID-19 daily update archives available on the DHHS public website [5].

## S5. DESCRIPTION OF SUPPLEMENTAL DATA

- Timeseries of total case incidence for the Crossroads Hotel and Cedar Meatworks studies
- Correlation values used in Figure 5(a), (b), and (c)
- 95% confidence intervals for Figures 5(a) and 5(b)

## S6. REFERENCES


[1] Schwartz J. Bing Maps Tile System; 2018. [Online; accessed 12-Aug-2020]. https://docs.microsoft.com/en-us/bingmaps/articles/bing-maps-tile-system.

[2] 1410.0 Data by Region, 2013-18; 2019. [Online; accessed 12-Aug-2020]. https://www.abs.gov.au/AUSSTATS/abs@.nsf/DetailsPage/1410.02013-18?OpenDocument.

[3] About TableBuilder; 2020. [Online; accessed 12-Aug-2020]. https://www.abs.gov.au/websitedbs/d3310114.nsf/home/about+tablebuilder.

[4] NSW COVID-19 cases by location and likely source of infection; 2020. [Online; accessed 11-Aug-2020]. https://data.nsw.gov.au/data/dataset/nsw-covid-19-cases-by-location-and-likely-source-of-infection.

[5] Updates about the outbreak of the coronavirus disease (COVID-19); 2020. [Online; accessed 11-Aug-2020]. https://www.dhhs.vic.gov.au/coronavirus/updates.
