# Supplementary material for: Risk mapping for COVID-19 outbreaks in Australia using mobility data
Source: J R Soc Interface. 2021 Jan 6;18(174):20200657. doi: 10.1098/rsif.2020.0657 (PMC7879754; doi:10.1098/rsif.2020.0657)
Supplement: Supplementary Technical Note [file rsif20200657supp2.pdf]

Technical note: interpolation of Origin-Destination mobility data between different geospatial partition schemes

Cameron Zachreson[1]

[1] *The University of Melbourne, Melbourne School of Engineering, School of Computing and Information Systems*

Abstract

Origin-destination matrices that represent the movement of populations between regions are ubiquitous data structures used frequently when building models of infectious disease transmission in mobile populations. Of course, the topology of such matrices depends on the classifications used to define regions (nodes) of the matrix. Typically the nodes of an origin-destination matrix are defined spatial regions, but any unique set of classifiers may be used to describe the flows of individuals between compartments. Often, it is necessary to interpolate data from one set of classifiers (regions) into a different, possibly overlapping, set. In this note, I describe a simple method and algorithm for performing this type of interpolation using correspondences represented as conditional probabilities of regional occupancy.

I. OVERVIEW

Given:

- set of N origins (O), these could be spatial regions, or any other appropriate classifier
- set of M destinations (D), this can be the same set of partitions used for origins, or it can be some other set of classifiers
- An $N \times M$ asymmetric, weighted adjacency matrix ($\mathbf{G}$), in which each element G_{ij} describes the movement of discrete quantities (i.e., populations) from origin node $i \in [1, N]$ to destination node $j \in [1, M]$
- set of L partitions (O^*) that will be the set of origins after re-partitioning
- set of K partitions (D^*) that will be the set of destinations after re-partitioning
- A correspondence $O \to O^*$. For example, a $N \times L$ matrix $\mathbf{P_O}$ of conditional probabilities $p_{nk}(O_k^* \mid O_n)$ describing the probability that an individual will be found in the k-th region of the set O^* given that they are in the n-th region of the set O.
- A correspondence $D \to D^*$, e.g., a $M \times K$ matrix $\mathbf{P_D}$ of conditional probabilities describing the correspondence between destinations (see above).

The method uses the correspondences $\mathbf{P_D}$ and $\mathbf{P_O}$ to re-partition the elements of $\mathbf{G}$ into a new $L \times K$ matrix $\mathbf{G}^*$.

II. METHODS

Once the partition sets and their correspondences are in hand, the method is straightforward and proceeds as per the example illustrated in Figure 1. Each connection between the new sets of partitions consists of a linear combination of components, one for each edge in the original matrix $\mathbf{G}$. For origin A and destination B in the original matrix $\mathbf{G}$, let $G(A \to B)$ represent the flow of individuals from A to B. For origin x and destination y in the new matrix $\mathbf{G}^*$, the contribution of $G(A \to B)$ is computed as follows:

$$G^*(x \to y) = \sum_{i,\ j} G(A_i \to B_j) \times P_O(A_i, x) \times P_D(B_j, y) \tag{1}$$

in which subscripts i, j indicate summation over all elements of $\mathbf{G}$ and $P_O(A, x) = p(x \mid A)$ and $P_D(B, y) = p(y \mid B)$ are the correspondences between origin and destination regions computed as conditional probabilities. The product $[P_O(A, x) \times P_D(B, y)]$ gives the probability that an individual departing from region A and arriving in region B also departed from region x to arrive in region y. Iterating over all pairs $G(A_i \to B_j)$ gives the set of factors composing each new connection. Of course, all elements for which $G(A_i \to B_j) = 0$, $P_O(A_i, x) = 0$, or $P_O(B_j, y) = 0$ may be omitted from the sum in implementation. In the MATLAB implementation included below, these exclusions are implemented implicitly in the sparse input tables, in which zero-valued entries are not included.

By expressing the correspondeces $\mathbf{P_O}$ and $\mathbf{P_D}$ as $N \times L$ and $M \times K$ matrices, respectively, the transformation $\mathbf{G} \to \mathbf{G}^*$, can be succinctly expressed as:

$$\mathbf{G}^* = \mathbf{P_O}^\top \, \mathbf{G} \, \mathbf{P_D} \tag{2}$$

While the method presented here is simple to implement, the production of the correspondences $\mathbf{P_O}$ and $\mathbf{P_D}$ can be non-trivial and will depend on the type of data represented in the matrix $\mathbf{G}$, as well as the types of compartments used for O, D, O^*, and D^*. As an example, consider the common case where O^* is a particular set of spatial partitions (e.g., administrative regions) and O is a dramatically different set of regions (e.g., Bing Tiles). To generate a correspondence, it is necessary to establish some type of overlap measure between these regions, this could be spatial, or it could be based on some other quantity that may vary in space (e.g., population), so that spatial overlap can be translated into the desired conditional occupancy probabilities. In the latter case, a useful technique is to find some set of partitions of much smaller scale, so that the quantities of interest (numbers of people, addresses, businesses, etc.) can be over-sampled for each region and the degree of overlap quantified, without requiring data on the level of individual people (which is typically not available).

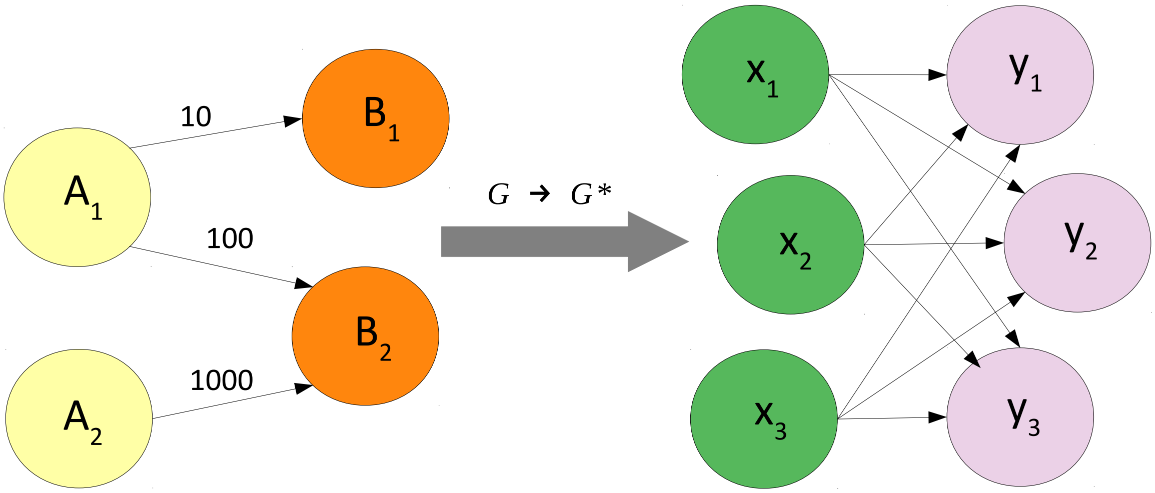

G	B_1	B_2
A_1	10	100
A_2	0	1000

P_O p(x|A)	x_1	x_2	x_3
A_1	0.5	0.4	0.1
A_2	0.5	0.2	0.3

P_D p(y|B)	y_1	y_2	y_3
B_1	0.4	0.3	0.3
B_2	0.1	0.3	0.6

G*	y_1	y_2	y_3
x_1	57	166.5	331.5
x_2	25.6	73.2	145.2
x_3	31.4	93.3	186.3

$$G*(x_{1,}y_1) = [G(A_{1,}B_1) \times P_O(A_{1,}x_1) \times P_D(B_{1,}y_1)] + [G(A_{1,}B_2) \times P_O(A_{1,}x_1) \times P_D(B_{2,}y_1)] + [G(A_{2,}B_1) \times P_O(A_{2,}x_1) \times P_D(B_{2,}y_1)] = 57$$

FIG. 1. Schematic of the described procedure

III. MATLAB IMPLEMENTATION

This code implements the example shown in Figure 1

```
% converts a matrix between partition schemes, ingredients are the
% orignial edge list and a correspondence file for conversion of boundaries
% between sets.

% input data structures

%input files are:

% OD_test.csv
% origin    destination     n
% A1        B1              10
% A1        B2              100
% A2        B2              1000

% PD.csv
% D_old  D_new  p
% B1     y1      0.4
% B1     y2      0.3
% B1     y3      0.3
% B2     y1      0.1
% B2     y2      0.3
% B2     y3      0.6

% PO.csv
% O_old  O_new   p
% A1     x1      0.5
% A1     x2      0.4
% A1     x3      0.1
% A2     x1      0.5
% A2     x2      0.2
% A2     x3      0.3

% original matrix, to be converted to new partition
% scheme -> table columns: {origin, destination, n}

input_filename = 'OD_test.csv';
output_filename = 'OD_out.csv';

% set up correspondence structures

```

```
% PO.csv -> table columns: {O_old, O_new, p}
% PD.csv -> table columns: {D_old, D_new, p}

PO_filename = 'PO.csv';
PD_filename = 'PD.csv';

PO_corr_table = readtable(PO_filename);
PD_corr_table = readtable(PD_filename);

orig_IDs = cellstr([ PO_corr_table.O_old ; PD_corr_table.D_old]);
new_IDs = cellstr([ PO_corr_table.O_new ; PD_corr_table.D_new]);
corr_vals = [PO_corr_table.p ; PD_corr_table.p];

corr_table = table(orig_IDs, new_IDs, corr_vals);

orig_ID_list = unique(corr_table.orig_IDs, 'rows');

% converting correspondence table into map of maps:
% outer key values will be old codes
% inner key values will be new codes
% inner values are the associated correspondence proportion

corr_map = containers.Map('KeyType', 'char', 'ValueType', 'any');

% initialise correspondence structure
for i = 1:size(orig_ID_list, 1)

    corr_map(orig_ID_list{i}) = containers.Map('KeyType', 'char', ...
        'ValueType', 'any');

end

% fill the inner maps

for i = 1:size(corr_table.orig_IDs, 1)

    id_source = corr_table.orig_IDs{i};
    tmp = corr_map(id_source);
    id_target = corr_table.new_IDs{i};
    corr_val = corr_table.corr_vals(i);
    tmp(id_target) = corr_val;
    corr_map(id_source) = tmp;

end

orig_edges = {};
```

```
e_table = readtable(input_filename);

for i = 1:size(e_table, 1)
    orig_edges{i, 1} = {e_table.origin{i}, e_table.destination{i}, ...
        double(e_table.n(i))};
end

% make the new edge list, each edge in the old list will map to edges ...
    in the
% new list based on the correspondence map.

new_edges = containers.Map('KeyType', 'char', 'ValueType', 'any');

% iterate through the old edge list, and distribute the commuters into the
% new edge list

% imperfect correspondence can lead to lost travellers,
% let's count them and see if it's a significant issue:
lost_travellers = 0;
total_travellers = sum(e_table.n);

for i = 1:size(orig_edges, 1)

    % each edge will produce a set of source and target nodes based on the
    % correspondence between partition schemes, these are the key ...
        values from
    % the inner corr_map associated with source and target codes

    old_source = orig_edges{i}{1};
    old_target = orig_edges{i}{2};
    w_old = orig_edges{i}{3};

    if ¬isKey(corr_map, old_source)
        lost_travellers = lost_travellers + w_old;
        fraction_lost = lost_travellers / total_travellers;
        fprintf(['no correspondence for tile ' old_source ', ',...
            '\n fraction travellers lost: ' num2str(fraction_lost) '\n'])

        continue

    end

    new_source_IDs = keys(corr_map(old_source));
    corr_source_old = corr_map(old_source);

```

```
    if ¬isKey(corr_map, old_target)
        lost_travellers = lost_travellers + w_old;
        fraction_lost = lost_travellers / total_travellers;
        fprintf(['no correspondence for tile ' old_target ', ',...
            '\n fraction travellers lost: ' num2str(fraction_lost) '\n'])

        continue

    end

    new_target_IDs = keys(corr_map(old_target));
    corr_target_old = corr_map(old_target);

    for j = 1:size(new_source_IDs, 2)

        if ¬isKey(new_edges, new_source_IDs{j})

            new_edges(new_source_IDs{j}) = ...
                containers.Map('KeyType', 'char', 'ValueType', 'any');
        end

        new_source_id = new_source_IDs{j};
        proportion_source = corr_source_old(new_source_id);
        tmp = new_edges(new_source_IDs{j}); %inner map

        for k = 1:size(new_target_IDs, 2)

            new_target_id = new_target_IDs{k};
            proportion_target = corr_target_old(new_target_id);
            w_new = w_old * proportion_source * proportion_target;

%             disp([old_source, ' , ', old_target, ', ' num2str(w_old)  ])
%             disp(['p_source: ' num2str(proportion_source), '; ... p_target: '...
%                 num2str(proportion_target)])
%             disp([new_source_id ' , ' new_target_id ' , ' ... num2str(w_new)])
%             disp(' ')

            if ¬isKey(tmp, new_target_IDs{k})

                tmp(new_target_IDs{k}) = w_new;

            else

                tmp(new_target_IDs{k}) = tmp(new_target_IDs{k}) + w_new;
```

```
            end
        end

        % update edge map
        new_edges(new_source_IDs{j}) = tmp;

    end
end

% convert edge map to table for export

source_ID_new = {};
target_ID_new = {};
edge_weight_new = [];

new_source_IDs = keys(new_edges);
edge_index = 0;

for i = 1:size(new_source_IDs, 2)

    target_IDs_i = keys(new_edges(new_source_IDs{i}));
    tmp = new_edges(new_source_IDs{i});

    for j = 1:size(target_IDs_i, 2)

        edge_index = edge_index + 1;
        source_ID_new{edge_index, 1} = new_source_IDs{i};
        target_ID_new{edge_index, 1} = target_IDs_i{j};
        edge_weight_new(edge_index, 1) = tmp(target_IDs_i{j});

    end

end

origin = source_ID_new;
destination = target_ID_new;
n = edge_weight_new;
new_edge_table = table(origin, destination, n);

writetable(new_edge_table, output_filename);


% OR, using the matrix implementation -
% note- this will cause memory issues if the matrices are large

```

```
G = [10, 100; 0, 1000]
P_O = [0.5, 0.4, 0.1; 0.5, 0.2, 0.3];
P_D = [0.4, 0.3, 0.3; 0.1, 0.3, 0.6];

G_star = P_O' * G * P_D
```
